# Integrated Moving on After Breast Cancer and Culturally Adapted Cognitive Behaviour Therapy Intervention for Depression and Anxiety Among Pakistani Women With Breast Cancer: Randomised Controlled Trial

**DOI:** 10.1192/bjo.2024.119

**Published:** 2024-08-01

**Authors:** Silsila Sherzad, Tayyeba Kiran, Imran Bashir Chaudhry, Nusrat Hussain, Nasim Chaudhry

**Affiliations:** 1 Pakistan Institute of Living and Learning, Quetta, Pakistan; 2 Balochistan Institute of Psychiatry and Behavioral Sciences, Quetta, Pakistan; 3 Pakistan Institute of Living and Learning, Islamabad, Pakistan; 4 Pakistan Institute of Living and Learning, Karachi, Pakistan; 5 University of Manchester, Manchester, United Kingdom

## Abstract

**Aims:**

Breast cancer is major cause of mortality in females, affecting 2.1m women annually. Annual mortality rates are double within south Asian women compared with high-income countries. Pakistan has very high rates of breast cancer. Co-morbid depression and anxiety is reported in more than one third of breast cancer survivors and predict higher recurrence and poorer survival.

Objective:

To evaluate the clinical effectiveness of Moving on After Breast Cancer Plus Cognitive Behaviour Therapy (Moving on ABC Plus) to reduce depression and anxiety in breast cancer survivors in Pakistan.

**Methods:**

A mixed method randomized controlled trial with 354 survivors of breast cancer recruited from the in- and out-patient services of oncology departments both from public and private hospitals of 5 major cities in Pakistan.

Individuals scoring 10 or higher on either the Patient Health Questionnaire-9 (PHQ-9) or the Generalized Anxiety Disorder scale (GAD-7) were enrolled. All participants underwent assessments using the PHQ-9, GAD-7, Functional Assessment of Cancer Therapy—Breast; EuroQol-5D; Multidimensional Scale for Perceived Social Support; Intrusive Thoughts Scale; and Rosenberg Self-Esteem Scale at the baseline and were randomly assigned to one of two trial groups: Moving on ABC plus or routine care. Those in the intervention group received 12 individual sessions of Moving on ABC plus, facilitated by trained master-level psychologists over 4 months. Follow-up assessments were conducted at 3- and 6-months after randomization. Individuals in routine care group continued their standard care. Qualitative interviews were conducted with 15 participants from the intervention group upon completion of the intervention.

**Results:**

The trial established the effectiveness of the integrated intervention, at 6-month follow up preserving 96% of retention. Intervention group reported a significantly higher reduction in depression, anxiety and intrusive thoughts, and improvement in health-related quality of life and self-esteem compared with routine care arm at end of the intervention. They endorsed the usefulness of intervention during qualitative interviews with improvement in psychological well-being, social support network, and interpersonal relationships. Fatigue was reported as a potential barrier to participating in the intervention.

**Conclusion:**

Results of this trial are in favour of psychological intervention; therefore, such programs should be implemented as part of routine care to reduce psychological distress and improve quality of life of this vulnerable population.

